# Safety of Ferric Carboxymaltose Immediately after Infliximab Administration, in a Single Session, in Inflammatory Bowel Disease Patients with Iron Deficiency: A Pilot Study

**DOI:** 10.1371/journal.pone.0128156

**Published:** 2015-05-26

**Authors:** Xavier Cortes, Joaquín Borrás-Blasco, Jose Ramón Molés, Maia Boscá, Ernesto Cortés

**Affiliations:** 1 IBD Unit, Gastroenterology Section, Internal Medicine Hospital of Sagunto, Sagunto, Spain; 2 University of Cardenal Herrera-CEU, Castellón, Spain; 3 Pharmacy Service, Hospital of Sagunto, Sagunto, Spain; 4 IBD Unit, Gastroenterology Department of the University Clinic Hospital of Valencia, Valencia, Spain; 5 Pharmacology, Paediatrics and Organic Chemistry Department, Miguel Hernández University, San Juan of Alicante, Spain; The Pennsylvania State University Hershey Medical Center, UNITED STATES

## Abstract

**Aim:**

To obtain preliminary safety and efficacy data on intravenous (IV) administration of infliximab (IFX) and ferric carboxymaltose (FCM) to inflammatory bowel disease (IBD) patients in a single treatment session.

**Methods:**

A two-phase non-interventional, observational, prospective pilot study was performed to evaluate safety and efficacy of FCM given immediately after IFX. IBD patients were recruited consecutively in the outpatient clinic in two groups. Control group patients (n = 12) received FCM on a separate day from IFX. Subsequently, single-session group patients (n = 33) received FCM after IFX on the same day. All patients received 5mg/kg IFX and 1000mg FCM for iron-restricted anemia (IRA) or 500mg FCM for iron deficiency without anemia. Safety assessment was performed by recording adverse events (AEs) during and immediately after infusion, 30 minutes afterwards, and via follow-up at 7 days and 8 weeks. For efficacy assessment, hematological parameters were assessed prior to FCM infusion (pre-FCM) and after 8 weeks. Economic impact of FCM given immediately after IFX was assessed.

**Results:**

All 45 patients (35 Crohn´s disease, 10 ulcerative colitis) received IFX 5mg/kg. 21 patients received 500mg FCM and 24 received 1000mg. FCM administration immediately after IFX corrected iron deficiency or IRA as shown by increases in hematological parameters. No AEs were reported during the safety evaluation at the end of FCM or IFX administration, 30 minutes, 7 days and 8 weeks afterwards, in either control or single-session groups. Total cost per patient for single-session administration was 354.63€; for patients receiving IFX and FCM on separate days, it was 531.94€, giving a 177.31€ per-patient cost saving.

**Conclusion:**

Single-session administration of FCM after IFX was safe and effective in IBD patients and can offer a good cost-benefit ratio and improve treatment adherence. To our knowledge, this study is the first to evaluate FCM and IFX administration in a single treatment session.

## Introduction

Inflammatory bowel disease (IBD) patients should be regularly assessed for the presence of anemia due to its high prevalence, its impact on quality of life, and comorbidity. Ulcerative colitis (UC) and Crohn’s disease (CD) are frequently accompanied by anemia. Anemia has been identified as a co-morbid condition that contributes to death in patients with IBD and it also increases the rate of hospitalization and medical costs for these patients [1]. The overall prevalence of anemia in patients with CD and UC has been estimated to be around 27% and 21%, respectively. Notably, 57% of the anemic patients were iron deficient [2].

The most common cause of anemia in IBD is iron deficiency, although other mechanisms might be relevant in some cases, such as high hepcidin levels due to inflammation, deficiency in vitamin B12, adverse events (AEs), hypersensitivity reactions and myelodysplastic syndromes. Iron deficiency is usually treated with oral iron supplementation, but many patients with IBD cannot tolerate oral iron preparations. Conversely, a large number of studies have shown a positive response to iron infusion in patients with IBD [3,4]. In 2009, a new parenteral iron drug was registered, ferric carboxymaltose (FCM) (Ferinject; Vifor Pharma, Switzerland). Using FCM, it is possible to administer 1000 mg iron during a single infusion, compared to only 200 mg which can be administered with iron sucrose (IS). The possibility of administering a single infusion of 1000 mg iron instead of 200 mg iron in each infusion with IS has positive implications for both the patient and the hospital, e.g. by reducing the number of venous punctures and hospital visits required, and potentially increasing patient compliance [5].

Recently, a European Consensus on the diagnosis and management of iron deficiency and anemia in IBD of the European Crohn´s and Colitis Organization (ECCO) was published. Iron-restricted anemia (IRA) is defined as any anemia where the erythropoietic activity is reduced due to the restricted availability of iron in the bone marrow, either by true iron deficiency or functional iron deficit. Intravenous iron should be recommended as first line treatment in patients with clinically active IBD, with previous intolerance to oral iron, with hemoglobin below 10 g/dl, and in patients who need erythropoiesis stimulating agents [6].

Infliximab (IFX) and ferric carboxymaltose (FCM) are of high value in IBD. Despite their frequent use in the treatment of IBD patients, there is a lack of evidence of their safety when administered in a single infusion session. Usually, two or more hospital visits are required to administer both treatments. The objective of this pilot study was to obtain preliminary safety data on administration of IFX (Remicade, MSD, Switzerland) and FCM in a single session in patients with IBD, in contrast to the usual clinical schedule of two or more hospital visits to administer both treatments. Patients were being treated with IFX for the combined management of the underlying disease and FCM for iron-restricted anemia or iron deficiency.

## Materials and Methods

We conducted a two-phase non-interventional, observational, and prospective pilot study to test the safety and efficacy of iron treatment using intravenous (IV) FCM immediately after IV IFX administration in adult patients diagnosed with IBD for at least 6 months being treated in an IBD gastroenterology (GI) outpatient clinic setting (day care hospital). The study was conducted at the Hospital of Sagunto, Spain in accordance with the Declaration of Helsinki and Good Clinical Practice guidelines and after approval of the protocol and its amendments by the local Ethics Committee.

Eligible IBD patients were 18 years or older being treated with IFX or scheduled to start IFX therapy, who had IRA (defined as Hb levels of < 13 g/dL in males, and < 12 g/dL in females) or evidence of iron deficiency (defined as serum ferritin < 100 μg/L and/or TSAT<20%) [6]. Further inclusion criteria were normal levels of vitamin B12 and folic acid. They had not received oral iron treatment agents for at least 8 weeks and had a documented poor tolerance or unresponsiveness to supplementation with oral iron. Patients were recruited consecutively in the GI outpatient clinic and gave signed informed consent.

For all IBD patients, clinical information was recorded, including type of IBD (CD or UC), disease pattern, extension and evolution of the disease, their current IBD treatment and previous use of anti–tumor necrosis factor-α agents or IV iron. The duration of the pilot study was 12 months.

Patients treated with IV or oral iron or blood transfusions in the 8 weeks prior to screening or who had a history of erythropoietin treatment were excluded. Further exclusion criteria were chronic alcohol abuse; chronic liver disease or increase in transaminases more than 3 times above the normal upper range limit; presence of portal hypertension with esophageal varices; known hypersensitivity to IFX or FCM; history of acquired iron overload; myelodysplastic syndrome; pregnancy or lactation; known active infection; clinically significant overt bleeding; active malignancy or chronic renal failure; surgery with relevant blood loss (Hb decrease>2g/dL) in the 3 months prior to the study; known human immunodeficiency virus; hepatitis B or hepatitis C virus infection; significant cardiovascular disease.

The FCM dose administered during the study was determined by the following criteria: IBD patients with IRA received 1000 mg FCM and IBD patients with iron deficiency only received 500mg FCM, at a maximal infusion time of 15 minutes. The higher dose of 1000mg FCM was consistent with the maximum single dose that could be administered in a single session according to the prescribing information for FCM [7]. The IFX administration dose was 5 mg/kg for all patients included in the study.

### Control group

In the first phase of the study, the control group was recruited, consisting of patients that received IFX and FCM on different days. The safety of IFX and FCM, as well as the efficacy of FCM, were assessed. Recruitment for the first phase of the study took place over 3 months.

### Single-session treatment group

In the second phase, patients received administration of IFX and FCM during a single session (single-session treatment group). They first received the IFX infusion, followed by a wash-out with 50 ml of normal saline and afterwards FCM was administered using the same venous access over a maximal infusion time of 15 minutes. Recruitment to the second phase of the study took place over 9 months.

### Laboratory and clinical data

In addition to demographic data and clinical disease characteristics, the IFX and FCM doses administered were recorded. Since the standard cycle for IFX treatment was 8 weeks, each patient received only one dose of both IFX and FCM during the 8-week study. In addition, relevant parameters were recorded for both treatment groups prior to the first FCM treatment (pre-FCM), and in the follow-up visit at 8 weeks (post-FCM).

Hematological parameters recorded were: hemoglobin (Hb), hematocrit, mean corpuscular volume (MCV), mean corpuscular Hb volume (MCH), red blood cell distribution width (RDW), leukocyte analysis, erythrocyte sedimentation rate (ESR), serum ferritin (s-ferritin), transferrin saturation (TSAT), total iron-binding capacity (TIBC) and serum iron.

To examine possible non-hematopoietic effects of FCM, C-reactive protein (CRP), as well as routine serum biochemical parameters before the first FCM infusion and during follow-up at 8 weeks were also determined.

In addition, IBD activity was measured according to the modified Truelove and Witts clinical classification of UC [8] and Harvey-Bradshaw Index for CD patients [9], prior to FCM and IFX infusion and at the 8-week follow-up visit.

### Safety evaluation

In order to identify any AEs associated with the drug infusion, a combination of the patient's response to questioning, physical examination, records of vital signs (BP, pulse, temperature) and laboratory parameters were evaluated. At the time of the indication of IV iron infusion (pre-FCM), a systematic medical history was taken. Pre-FCM, immediately after the infusion of both IFX and FCM, 30 minutes afterwards, and at the 8-week follow-up visit (post-FCM), patients were subject to questioning, physical examination and methodical measurement of vital signs. In addition, patients were questioned during a follow-up telephone call 7 days after the FCM infusion. All AE evaluations were performed following a structured questionnaire which included IFX and FCM adverse drug reactions cited in the respective prescribing information for both products: nausea, headache, dizziness, fever, urticaria, infusion-site reactions, nervous system disorders, cardiac arrhythmia or myocardial ischemia, hypertensive or hypotensive events, skin reactions, gastrointestinal disorders, infections, hypersensitivity or anaphylaxis. Furthermore, patients were asked open questions about their general wellbeing in order to solicit the reporting of any other AEs.

Laboratory parameters including white blood count, liver transaminase levels, phosphate levels and renal function were studied pre-FCM treatment and at the 8-week follow up (post-FCM).

The assessment of procedure-related safety was performed by continuous monitoring by the gastroenterologist in charge of the infusion. AEs including potential local and systemic adverse effects were evaluated separately considering those during infusion, those reported 30 minutes after the end of infusion and monitoring 7 days and 8 weeks after infusion. In order to assess the causality of any AEs related to IFX and FCM, the Naranjo ADR probability scale was applied [10].

### Outcome measures

Efficacy outcomes related to FCM treatment in the two groups were defined differently for patients with IRA than for those with iron deficiency. For IRA patients, efficacy outcomes were defined as an Hb increase of at least 2 g/dL (≥2g/dL) and/or normalization of Hb (to Hb >12 g/dL in females, Hb >13 g/dL in males) over the 8-week study period. Efficacy outcomes for patients with iron deficiency were TSAT normalization (TSAT >20%), or an increase in s-ferritin to >100 μg/L over the 8-week study period.

### Cost evaluation

For 2 sessions at the GI outpatient clinic (day care hospital) on separate days, the total cost of IV treatment (IFX, FCM) was 531.94€ per patient. The total cost of IV treatment (IFX, FCM) administration on the same day for both drugs given in one session was 354.63€ per patient. Administration cost data was taken from the 2014 General Price Index in the S.I.E. (Sistema de Información Económica) Catalogo de Hospital de Día of the Hospital de Sagunto (Conselleria Sanitat Comunitat Valenciana, Spain) [11].

### Statistical analysis

Normally-distributed continuous variables were described as mean ± standard deviation (SD) and comparisons between pre- and post-FCM administration in the two groups were performed using a paired sample T-test. For variables which were not normally distributed, values were expressed as median with the interquartile range (IQR), and the Mann–Whitney test and Wilcoxon Signed Rank test were performed. Chi square analysis was used when comparing frequencies. For correlation, the Spearman Rank correlation was used. A p-value of 0.05 (two-tailed) was considered to be significant. Statistical analysis was conducted using the SPPS 19.0 working package (SPSS Inc., Chicago, IL).

## Results

45 consecutive patients (35 with Crohn´s Disease and 10 with Ulcerative Colitis) were included.

No AEs were reported in either group (control or single-session) at the end of the IV administration (both IFX and FCM), irrespective of whether the IV administration of both drugs occurred on separate days or in a single session. Furthermore, no AEs were reported 30 minutes after completion of the infusion, 7 days afterwards or at the 8-week follow-up safety evaluation in the GI outpatient clinic. There were no treatment-related anaphylactic reactions, no infusion site reactions and no clinically-significant changes in laboratory parameters.

### Control group

Twelve consecutive patients (mean age 45.8±15.2 years; 4 women), 8 patients with CD and 4 patients with UC, were included. All patients received an IFX dose of 5 mg/kg. Four patients received an infusion of 500 mg FCM and 8 patients received 1000 mg (Table 1). The cumulative IV administration time (IFX plus FCM) was on average 3.5 hours, when IFX and FCM were administered on separate days.

**Table 1:** Demographic and clinical characteristics of IBD patients in the control group and the single-session treatment group.

	Control group (n = 12)	Single-session treatment group (n = 33)
**Sex (M/F)**	8/4	16/17
**Age (years, mean±SD)**	45.8±15.2	40.1±12.8
**Weight (kg, mean±SD)**	65.5±10.2	65.7±12.9
**Crohn´s disease/ulcerative colitis**	8/4	27/6
**Iron deficiency/ iron-restricted anemia**	8/4	13/20
**Ferric carboxymaltose dose (500mg/1000mg)**	8/4	13/20
**Infliximab dose**	5 mg/kg	5 mg/kg
**Number of visits to GI outpatient clinic for IV treatment administration (IFX, FCM)**	2	1
**Infliximab patient (naïve/pretreated)**	2/10	8/25
**Years of IBD diagnosis (mean±SD)**	7.8±9.2	12.5±10.5
**Active IBD** * **(Y/N) pre-FCM**	11/1	28/5
**Active IBD** * **(Y/N) post-FCM**	9/3	20/13
**Mean CRP (pre-FCM/post-FCM)**	10.1/14.6	18.4/8.6
**Treatment successful** ** **(Y/N)**	8/4	26/7
**Adverse Drug Reactions**	None	None

(*) As defined by the modified Truelove and Witts index [8] and Harvey-Bradshaw Index [9]

(**) Successful treatment was defined for iron-restricted anemia as Hb increase ≥2 g/dL or Hb normalization (Hb >12 g/dL in female, Hb >13 g/dL in male patients), and for iron deficiency as TSAT normalization (TSAT >20%) and/or s-ferritin >100 μg/L, over the 8-week study period.

**Fig 1:**
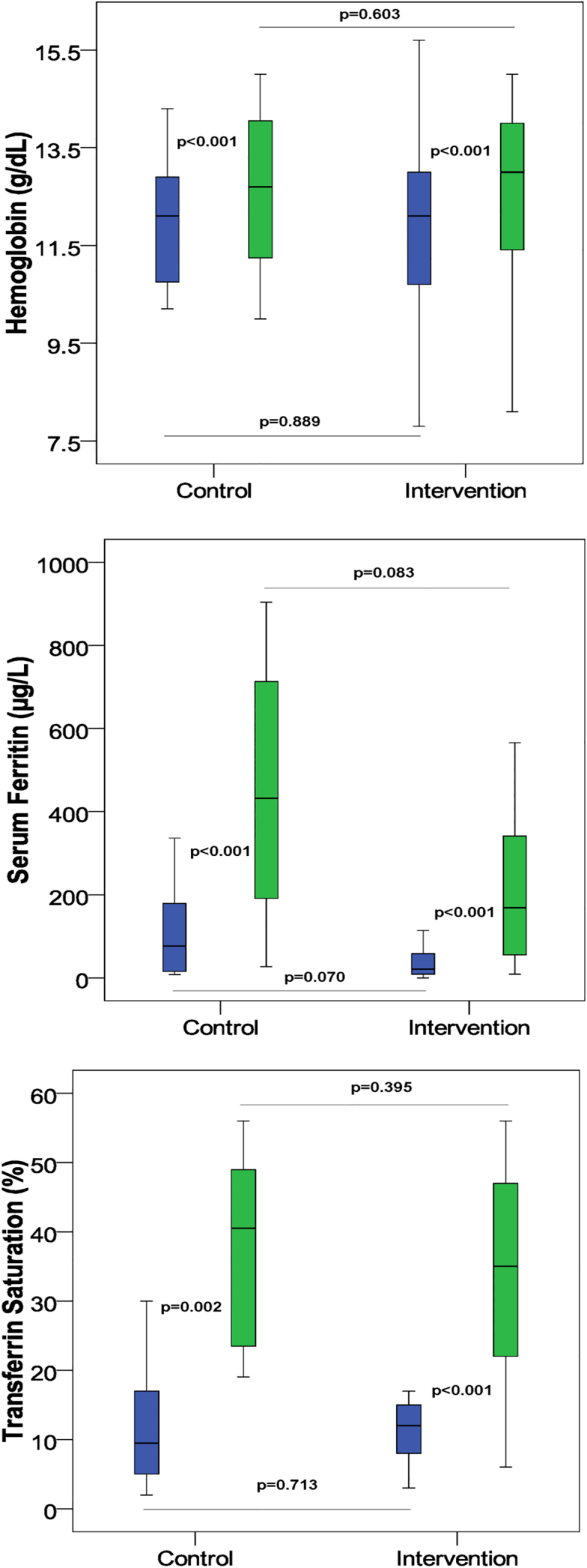
Hemoglobin, serum ferritin and transferrin saturation levels of the control group (control) and the single-session group (intervention) in IBD patients pre- and post-FCM (ferric carboxymaltose) treatment. Blue color: pre-FCM and green color: post-FCM. The horizontal line represents the median, the box-plot represents the interval of the interquartile range (Quartile 1-Quartile 3).

After eight weeks in the control group, the mean hemoglobin levels increased from 11.8±1.6 to 12.6±1.6 g/dL (p<0.001) (Fig 1), the mean hematocrit increased from 36.7±5.5 to 38.7±4.4% (p<0.005). The mean s-ferritin increased from 141.0±195.2 to 438.0±311.8 μg/L (p<0.001) (Fig 1), and the TSAT from 11.8±8.7 to 38.2±13.4% (p<0.001) (Fig 1). Major hematologic parameters (mean±SD) pre- and post-FCM infusion are shown in Table 2.

**Table 2:** Major hematologic parameters pre- and post-ferric carboxymaltose infusion.

	Control group (n = 12)			Single-session treatment group (n = 33)		
	Pre-FCM	Post-FCM (after 8 weeks)	p value	Pre-FCM	Post-FCM (after 8 weeks)	p value
**Hb (g/dl)**	11.8(1.9)	12.6(1.6)	<0.001	11.7(2.2)	13.3(3.5)	0.006
**HCT (%)**	36.7(5.5)	38.7(4.4)	0.001	36.0(6.4)	38.6(4.9)	0.003
**s-ferritin (μg/l)**	141.0(195.2)	438(311.8)	<0.001	63(103.6)	274.9(324.8)	<0.001
**TSAT (%)**	11.8(8.7)	38.2(13.4)	<0.001	11.4(4.1)	35.2(21.1)	<0.001
**MCV**	79.3(11.0)	86.6(11.9)	0.005	85.8(10.9)	90.7(9.8)	<0.001
**MCH**	26.2(4.2)	32.4(19.8)	0.099	28.0(4.1)	30.0(3.5)	<0.001
**RDW**	17.2(3.0)	16.5(2.9)	0.326	16.2(3.1)	17.2(4.0)	0.212

Values are expressed as mean (standard deviation). P-values were calculated using the Wilcoxon Signed Rank test.

At week 8, treatment success was 67% with 8 out of 12 patients in the control group showing either normalization of Hb or Hb increase ≥2 g/dL, or normalization TSAT or/and s-ferritin (Table 1).

Total costs associated with the IV infusion of IFX and FCM on a different day for the 12 patients included in the study were 6,383€. Mean administration cost per patient was therefore 531.94€.

### Single-session treatment group

Thirty-three consecutive patients (mean age 40.1±12.8 years; 17 women) comprising 27 CD patients and 6 UC patients were included in the single-session treatment group. All patients received an IFX dose of 5 mg/kg. Thirteen patients received an infusion of 500mg FCM and 20 patients received 1000 mg (Table 1). The mean administration time for IFX followed by FCM was 2.5 hours. After 8 weeks, the mean Hb levels increased from 11.7±2.2 to 13.3±3.5 g/dL (p<0.006) (Fig 1). Furthermore, there were significant increases in mean hematocrit (from 36.0±6.4 to 38.6±4.9%, p<0.003), mean s-ferritin (from 63.0±103.6 to 274.9±324.8 μg/L, p<0.001) and TSAT (from 11.4±4.1 to 35.2±21.1%, p<0.001) (Fig 1). At week 8, treatment success was 79% with 26 out of 33 patients having either normalized Hb levels or Hb increase ≥2 g/dL, or normalization TSAT or/and s-ferritin (Table 1). Major hematologic parameters (mean±SD) pre- and post-FCM infusion are shown in Table 2.

Total costs associated with the IV infusion of IFX followed by FCM in the same session for the 33 patients included in the study were 11,702€, giving a mean cost of administration per patient of 354.63€.

As expected, both study groups showed similar gains in Hb, s-ferritin and TSAT over the 8-week study period, and there were no statistically significant differences between control and single-session treatment groups for any of these efficacy parameters (Fig 1).

## Discussion

Iron deficiency is the main cause of anemia in IBD and responds well to iron-replacement therapy. Iron supplementation should be considered in every patient suffering from iron deficiency with or without anemia. Intravenous iron therapy is more effective, has a faster and higher response rate, and it is better tolerated by patients, showing a lower discontinuation rate in IBD patients due to adverse events than oral iron supplementation[12,13].

FCM is an IV iron preparation that can be administered at single doses of up to 1000 mg iron within 15 minutes. The efficacy and tolerability of FCM has been shown in various indications, including iron deficiency or in anemia associated with IBD [14]. This new IV iron formulation reduces both the risk of free iron reactions as well as that of immunogenicity. Infusion treatment duration is only 15 minutes for a 1000mg dose compared with longer administration time of other IV formulations such as iron sucrose (IS), ferric gluconate, or low molecular weight iron dextrans. Evstatiev *et al* showed in a randomized, controlled, open-label, multicenter study that the simpler FCM-based dosing regimen showed better efficacy and compliance, as well as a good safety profile, compared to the Ganzoni-calculated iron sucrose dose regimen [15].

Furthermore, FCM administration presents advantages from an economic perspective. Calvet *et al* performed a cost-minimization analysis to compare the cost impact of FCM and IS in patients with severe iron deficiency treated at a GI outpatient clinic. The results of the study showed that the estimated direct hospital costs for iron infusion per patient per year was 304€ for IS and 274€ for FCM, a difference of 30€ in favor of FCM. Adding non-hospital direct costs increased the difference to 67€ [16].

The abovementioned study showed that IFX followed by FCM could be successfully administered on a different day with a good tolerance and overall safety. Based on the data, we considered studying the sequential administration of IFX and FCM on the same day, in the same infusion session. The results of this pilot study demonstrate for the first time that IFX followed by FCM can be successfully administered showing good tolerance and overall safety profile. No adverse drug event was seen after 8 weeks of follow-up and no infusion-related adverse events or anaphylactic reactions were reported.

This study also demonstrated that in patients with IBD and either iron-restricted anemia or iron deficiency, FCM administered immediately after IFX in a single session was able to correct both functional iron deficiency as well as true iron deficiency in the majority of patients, as reflected by increases in Hb, hematocrit, s-ferritin and TSAT, markers which are recommended by the ECCO guidelines to show resolution of anemia and normalization of iron stores [6]. Furthermore, we saw similar efficacy in our study when compared to previous studies [15], even though patients' inflammatory activity was high and remained elevated, as determined by high CRP levels and IBD activity index measured pre- and post-FCM (Table 1).

Administration of FCM immediately after IFX treatment in a single session may have additional benefits, including more convenience for the patient due to fewer visits to the outpatient clinic and consequently a decrease in lost working hours, reducing patient waiting time and travel costs, better optimization of outpatient resources and a reduced waiting list, together with decreased IBD patient care costs for the hospital. In current clinical practice the administration of IFX and FCM is scheduled for separate sessions on different days. This administration strategy has several disadvantages: the patient must come to the GI outpatient clinic twice (on different days) placing a higher burden on hospital resources and resulting in lower patient comfort; more frequent hospital visits can lead to increased stress for the patient; therefore, it is a more troublesome and expensive strategy for the patient. In order to improve patient comfort, optimize health resources and decrease drug administration cost, we scheduled our IBD patients to receive IFX and FCM in the same session at our GI outpatient clinic.

Data on the administration of IFX and IV iron to IBD patients in a single session is scarce. Katsanos *et al*. assessed the safety of IV IS [Venofer] given immediately after IFX treatment in 37 IBD patients. IS was scheduled on the same day (dose 200–400 mg), immediately after IFX infusion. Administration of both drugs over the course of the whole 12-week treatment cycle resulted in significant increases in EPO and soluble transferrin receptors (sTFR) compared to baseline pre-IFX levels. Twenty-three out of 29 patients (79%) with CD receiving systemic IFX therapy (5mg/kg at 8-week intervals) had Hb ≥12 g/dL after receiving IV IS during the study. AEs considered possibly or probably related to IV IS administration were recorded in 2 female patients (2.3% of patients, 0.7% of infusions) both in IFX episodic therapy. One patient had nausea and skin rash and the other patient developed diffuse urticaria. A further patient was hospitalized with a diagnosis of a lower respiratory tract infection or delayed hypersensitivity to episodic IFX [17].

Following the safety criteria in the FCM prescribing information [7] we administered the maximum single dose of FCM (1000mg) recommended for IBD patients with anemia, and 500 mg for IBD patients with iron deficiency, after IFX administration.

Our study showed that administration in a single session resulted in considerable cost savings. For a patient treated in the same session with IFX followed by FCM, the cost was 354.63€, whereas the cost of IFX and FCM administrated on different days was 531.94€. Therefore, the cost savings per patient was 177.31€ and it implies substantial hospital savings.

Although it was prospective, only limited conclusions can be drawn from the study since it had a small sample size and only direct hospital administration drug costs were taken into account. For a more comprehensive overview, indirect costs and costs for the patient, such as travel or lost work hours would also need to be taken into consideration [18].

## Conclusion

Sequential IV administration of FCM following IFX treatment in one day in a single session was safe, well tolerated and effective in IBD patients, reducing costs for both the hospital and patients. In comparison to the common clinical practice of infusing IFX and iron on separate days, this single-session regimen could offer a good cost-benefit ratio and at the same time improve treatment adherence and patients’ quality of life.

To our knowledge this pilot study is the first to evaluate the efficacy and safety of administration of IFX followed by FCM in the same treatment session. In order to confirm our results, further controlled prospective studies with a larger patient population are needed.
